# Noninvasive paternal exclusion testing for cystic fibrosis in the first five to eight weeks of gestation

**DOI:** 10.1038/s41598-018-34396-6

**Published:** 2018-10-29

**Authors:** David A. Zeevi, Fouad Zahdeh, Yehuda Kling, Tzvia Rosen, Paul Renbaum, Raphael Ron-El, Talia Eldar-Geva, Hananel E. G. Holzer, Ephrat Levy-Lahad, Gheona Altarescu

**Affiliations:** 1 Medical Genetics Institute, Shaare Zedek Medical Center, Jerusalem, Israel; 2 Infertility and IVF Unit, Assaf Harofeh Medical Center, Zerifin, Israel; 3 IVF Unit, Division of Obstetrics and Gynecology, Shaare Zedek Medical Center, Jerusalem, Israel; 4 Hebrew University Faculty of Medicine, Jerusalem, Israel

## Abstract

Prenatal genetic testing is not generally applicable to the very early stages of pregnancy (prior to week 8 gestation), a time period that is crucial to pregnant couples with high risk for transmission of genetic disease to their fetus. Therefore, we developed a new ultra-sensitive targeted next generation sequencing method for noninvasive haplotype-based paternal allele exclusion testing of the cystic fibrosis-associated gene, *CFTR*. This new method was compared to a conventional library prep and sequencing analysis method and all test results were validated by amniotic fluid testing at later stages of pregnancy. Out of 7 enrolled couples, who provided at least two blood samples (at least one week apart) for noninvasive *CFTR* testing, a result was obtained for 6 fetuses. Using the new hypersensitive method, all six couples (100%) received a correct diagnosis for the paternal allele as opposed to 3/6 (50%) when tested with the conventional strategy. Among 4 couples who provided just one early pregnancy blood draw for analysis, diagnosis was possible in one fetus, but only using the ultra-sensitive method. Thus, we describe a novel noninvasive *CFTR* screening method which demonstrates unprecedented fetal allele typing accuracy in the earliest stages of pregnancy.

## Introduction

Fetal testing at very early gestational age remains one of the last frontiers in the burgeoning field of prenatal diagnosis nowadays. Although pregnant couples currently have an assortment of tools and screens to detect malformations and genetic disorders in their developing fetuses, these are only available from 10 weeks of pregnancy at the earliest. Standard prenatal testing tools include invasive and noninvasive tests, genetic and anatomical tests, and high risk and low risk pregnancy tests. Of course, there are advantages and disadvantages to each of these screening/testing methods in terms of scope and accuracy regarding the prediction of phenotypes in the fetus. Nonetheless, all current prenatal diagnostics are hampered by harsh obstacles relating to early pregnancy fetal testing^1–4^.

Genetic tests, in particular, are not well applied in the very early stages of pregnancy (prior to week 8 gestation). Invasive tests, such as chorionic villus sampling (CVS) and amniocentesis, are not safe to perform before week 11^1,5^; and much safer noninvasive tests, such as aneuploidy screen noninvasive prenatal testing (NIPT), are not generally accurate before week 8^6,7^. This leaves a small, yet crucial, time period in pregnancy (namely, weeks 4 through 8 gestation) during which assessment of the genetic status of a fetus is not generally feasible. This very early pregnancy time period may be critical mainly to pregnant couples with high risk for transmission of genetic disease^6,8^.

In this study, we investigated the prospect of very early prenatal diagnosis by performing weeks 5 through 8 pregnancy noninvasive prenatal screening for cystic fibrosis (CF). We chose CF for proof of principle testing due to several factors: 1) the carrier status is high in many worldwide populations; 2) it is one of the most commonly tested single gene disorders among expectant couples worldwide^9^; and 3) in the locale of our medical center, CF-causing founder mutations and founder haplotypes are prevalent in the local populace^10^. One of the aims of this project was to develop a genetic test with sufficiently rapid turnaround time to allow expectant couples to reach antenatal decisions regarding their fetus prior to week 9 gestation. Thus we established a one week regimen, from venipuncture to test result, in which we further streamlined our previously described noninvasive screening assay^11^ to facilitate prompt sample processing of early pregnancy samples. Finally, very early pregnancy noninvasive screening requires utmost sensitivity without compromising on specificity to prevent unwanted false positive test results. Accordingly, we describe a new ultra-sensitive high throughput genetic testing method which we developed for applied testing on a sizable cohort of early pregnancy couples (11 altogether) for paternal allele classification of the CF-associated genomic region. Our results suggest that although very early pregnancy genetic testing is challenging, our new methodological approach is appreciably more reliable than existing low and high throughput noninvasive testing methods at the earliest stages of pregnancy.

## Results

### Study description

Eleven pregnant couples were recruited into the study. Five of the couples achieved pregnancy via preimplantation genetic diagnosis (PGD) for cystic fibrosis (CF) and the rest had performed PGD for other genetic disorders and consented to allow their early pregnancy plasma samples to be used for allelic inheritance testing of intronic or gene-flanking *CFTR* single nucleotide polymorphisms (SNPs). In all cases, maternal plasma samples were collected during early stages of pregnancy (spanning from week 5 until week 8 gestation according to the date of embryo transfer) and the genetic status of the *CFTR* locus in each fetus was not known to the investigators at the time of plasma testing. For CF PGD couples, since only unaffected embryos are returnable, bi-allelic loss-of-function mutations in the *CFTR* gene (compound heterozygous or homozygous) were not expected to be in the fetus. However, given the policy in our institution that carrier embryos for recessive monogenic disorders are transferable, discrimination between paternal carrier state, maternal carrier state, and wild type scenarios could not be anticipated ahead of time. Nonetheless, as a proof-of-principle study and due to the very early pregnancy week of testing, the focus of this investigation was to assess only the paternal allele in the fetus which had a 66% likelihood to be wild type or 33% likelihood to be mutant in CF-PGD cases; or 50% likelihood to be reference or alternate SNP states in non-CF PGD cases. Validation of plasma test results was accomplished by testing the *CFTR* mutation/variant of interest in amniotic fluid DNA from each respective pregnancy.

### *In vitro* simulation of paternal allele identification in early pregnancy fetuses

As a key preliminary step in the process, we first sought to establish a sensitive technique for paternal CF allele assessment in very early pregnancy plasma samples. Currently accepted practice in the noninvasive prenatal diagnosis (NIPD) field is not to perform Mendelian disorder testing prior to week 9 gestation^12^. The primary obstacle to mutation testing before this time is low fetal fraction which, prior to week 8 gestation, rarely rises above the widely reported 4% lower threshold for effective NIPT diagnosis^13–16^. When fetal fraction is below 4% it has been extremely difficult to discriminate ‘background noise’ of sequencing or digital PCR errors from true biological events, such as wild type or mutant allele transmission to a fetus, at such prohibitively low fetal dosages^13,14,16^. For this reason, we developed an ultra-sensitive method, termed allele sensitive proliferation sequencing (ASP-SEQ), for diffuse molecule detection even at dosages well below 0.5% where fetal DNA concentration cannot be reliably measured.

**Figure 1:**
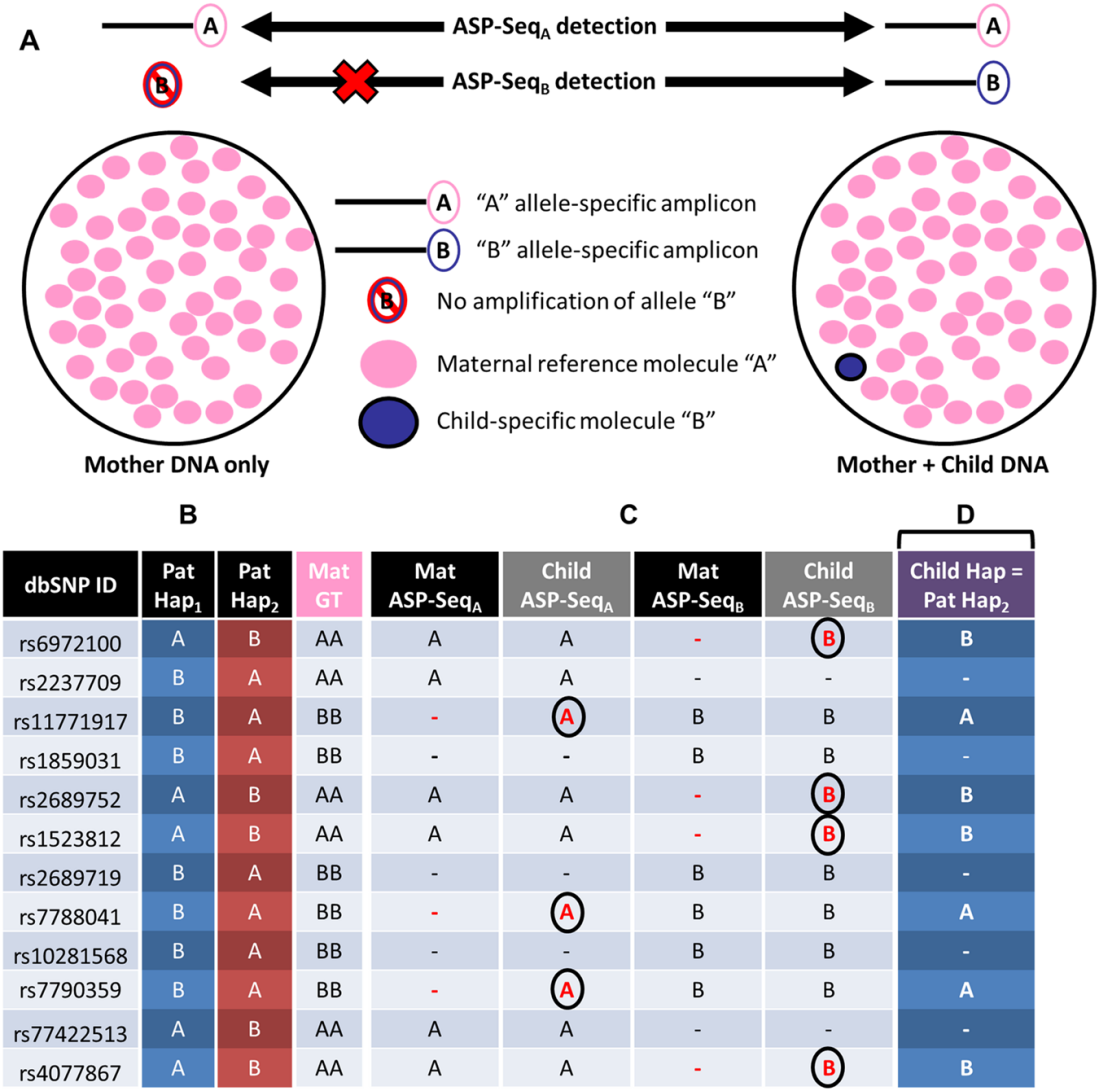
ASP-SEQ is designed to detect “low dosage” alleles with high sensitivity. (**A**) Illustration of basic ASP-SEQ principles. For every maternal genomic and accompanying plasma DNA sample with standard deep sequencing genotype information, two different targeted ASP-SEQ libraries are prepared. ASP-Seq_A_ libraries amplify only reference SNP alleles (“A”) but not non-reference alleles (“B”). Conversely, ASP-Seq_B_ libraries amplify only non-reference SNP alleles (“B”) but not reference alleles (“A”). After high throughput sequencing of each ASP-SEQ library, successfully amplified regions are mapped to the human genome and utilized to detect fetal DNA (illustrated as “Mother + Child DNA” in right-most circle) that does not exist in maternal-only genomic DNA (illustrated as “Mother DNA only” in left-most circle). Thus, for every fetal haplotype informative SNP locus, ASP-SEQ will determine whether a “child-specific” allele was transmitted to the fetus or not. In the pictured example, reference SNP allele “A” was successfully amplified by ASP-Seq_A_ libraries from both “Mother only” and “Mother + Child” plasma DNA samples; while ASP-Seq_B_ libraries only amplified allele “B” in “Mother + Child” plasma but not “Mother only” DNA. This ASP-SEQ detection pattern clearly indicates that the fetus inherited the “B” allele. Note that ASP-SEQ is especially designed to detect child-specific DNA molecules even though they are heavily diluted in maternal DNA (as in the right-most circle). (**B**) In a typical ASP-SEQ experiment, paternal haplotype informative heterozygous SNP loci are deduced from maternal and paternal haplotype-phased high throughput sequencing information. Relevant SNP loci are then organized by dbSNP ID, phased paternal haplotypes (“Pat Hap_1_” and “Pat Hap_2_”), and maternal genotype (“Mat GT”). (**C**) In parallel, ASP-SEQ is performed separately on plasma DNA and genomic DNA of the pregnant index and, for every haplotype informative SNP, ASP-SEQ output is tabulated in maternal-only genomic DNA ASP-SEQ libraries (“Mat ASP-Seq_A_” and “Mat ASP-Seq_B_”) and maternal + child/fetus plasma ASP-SEQ libraries (“Child ASP-Seq_A_” and “Child ASP-Seq_B_”). Child/Fetus haplotype informative alleles are circled and in red font. (**D**) Child/Fetus haplotype information derived from (**C**) was compared to paternal haplotype information in (**B**). In this example, the child/fetus had clearly inherited paternal haplotype 2 (“Child Hap = Pat Hap_2_”).

**Figure 2:**
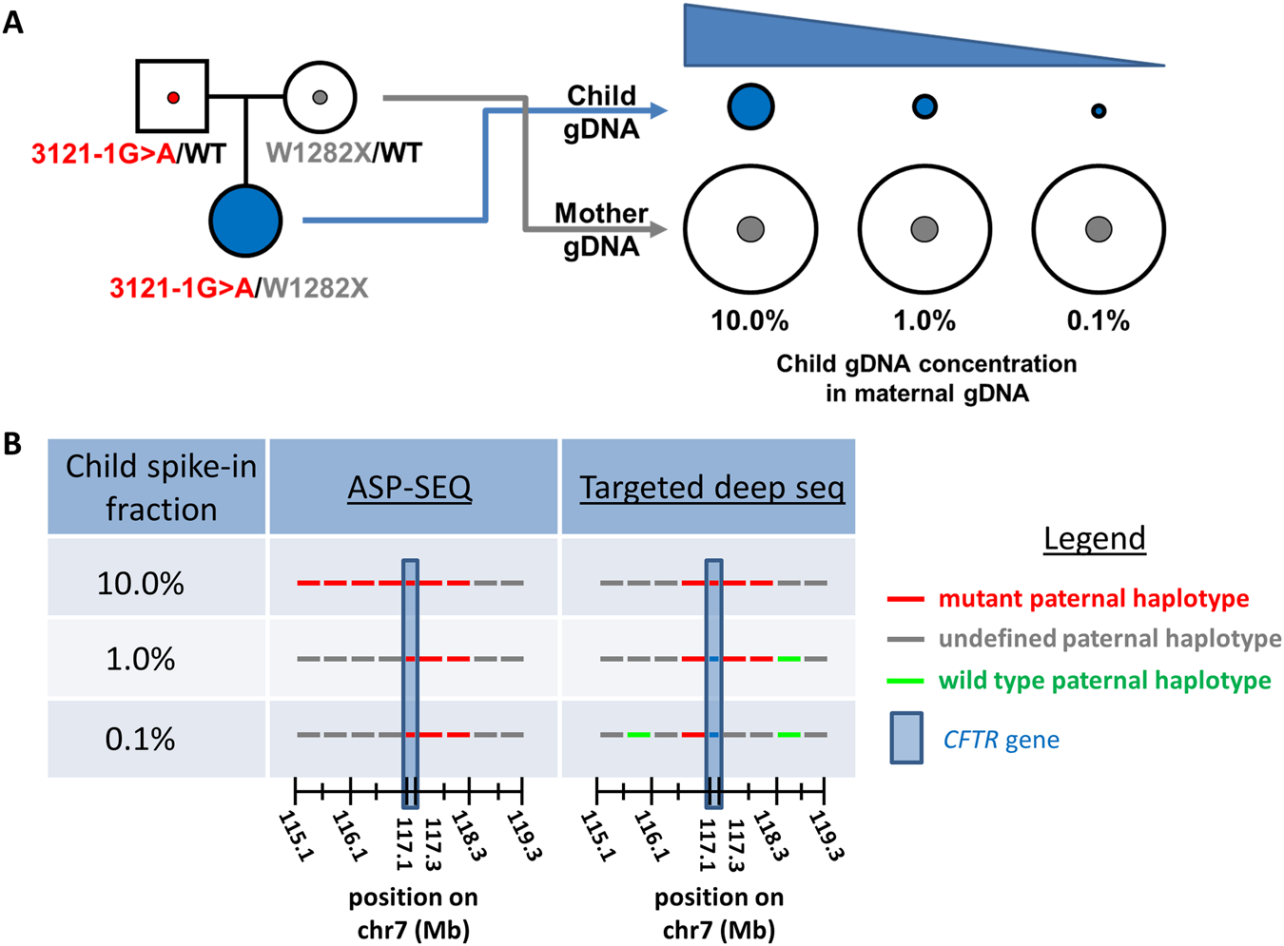
ASP-SEQ outperforms targeted deep sequencing (TDS) in an early pregnancy NIPD simulation. (**A**) Genomic DNA from a trio (Family ‘A’) of *CFTR* mutation carrying individuals was selected for NIPD simulation testing. Both mother and child DNA were sheared to typical plasma DNA size (~150–220 bps) and the resultant sheared child DNA was then diluted into various sheared mother DNA tubes at the three indicated ‘child dosage’ concentrations. Each mother-child mix was then diluted to 100 pg/ul DNA concentration followed by TDS and ASP-SEQ library prep, respectively. In addition, TDS was performed on bulk DNA samples of the Family ‘A’ trio for haplotype classification and test validation; and maternal bulk DNA was processed for ASP-SEQ as described in Fig. 1. (**B**) Results of the simulation in (A) are depicted as *CFTR* gene-flanking (+/−2 Mb; hg19 reference genome) paternal haplotype block predictions of ASP-SEQ and TDS (‘Targeted deep seq’) for each mother-child spike-in experiment as indicated in the Legend. Note that only ASP-SEQ correctly identified paternal mutant haplotype blocks in all mother-child mixes (even at child DNA dosage as low as 0.1%) while TDS provided consistent haplotype classifications solely in the highest (10%) child dosage sample.

ASP-SEQ is a new high throughput genotyping methodology (described in Fig. 1) aimed at detecting highly dilute paternal alleles in maternal plasma with high diagnostic confidence. For preliminary testing of this method, we devised a NIPD simulation using DNA samples from a CF PGD family in our clinic, family ‘A’, comprised of a couple and their CF-affected daughter (Fig. 2A). Each parent happened to carry a different disease-causing *CFTR* mutation but, unlike many conventional NIPD genotyping methods, ASP-SEQ is not limited to such scenarios where the mother and father of a fetus are carriers of differing mutations. To simulate the minuscule amounts of fetal DNA in plasma of a pregnant woman, peripheral blood DNA of the family ‘A’ mother and child were both sheared to typical plasma DNA size (ranging from 150 bp to 220 bp) in separate tubes. Subsequently, the sheared child DNA was spiked into the sheared mother DNA at 10.0%, 1.0%, and 0.1% dosages, also each in separate tubes (Fig. 2A). The resulting mother-child DNA mixtures were then diluted to 100 pg/ul concentration to simulate relatively low plasma DNA extract concentration and ASP-SEQ was performed on each mixture. For comparison with existing NIPD methods, we also performed targeted deep sequencing of 1,700 *CFTR*-flanking SNPs on each mother-child mixture and all haplotype designations were validated by deep sequencing of bulk DNA samples from each family ‘A’ individual. As mentioned above, 4% fetal dosage has typically represented the lower threshold for effective paternal mutation NIPD with existing technologies. Therefore, it was not surprising that both ASP-SEQ and targeted deep sequencing (TDS) effectively classified the paternal 3121-1 G>A mutation in the 10% child dosage mixture (Fig. 2B). However, at 1% child dosage (below the 4% threshold), TDS returned false haplotype classification in the 3′ genomic region downstream to *CFTR*; and at 0.1% child dosage, TDS returned false haplotype classification in both the 5′ and 3′ *CFTR*-flanking genomic regions. In contrast, our new ASP-SEQ method returned 100% correct mutant haplotype classification in all child dosage mixtures, including the technically challenging 0.1% child dosage (Fig. 2B). Thus ASP-SEQ is the preferred method for extremely low dosage paternal allele detection in maternal blood with potential for application in very early pregnancy plasma testing.

### Paternal allele identification in early pregnancy plasma samples

After demonstrating ASP-SEQ effectiveness in a model system, we further challenged the technique with ‘live’ early pregnancy plasma samples from our 11 couple study cohort. Of the 11 couples, 5 were carriers of CF mutations and the others were tested for allelic transmission of intronic or gene-flanking *CFTR* SNPs (see Table 1). Seven couples provided two or more early pregnancy plasma samples for testing while the other four couples provided only one plasma sample each. In all cases, paternal inheritance was tested by ASP-SEQ in pregnant indexes at different time points ranging from week 5 through week 8 gestation. Here too, we used TDS as a conventional NIPD technique for comparison. All assayed fetal dosages, NIPD results, subsequent amniotic fluid testing results, and other details from the ‘live’ early pregnancy study are summarized in Table 1.

**Table 1:** Summary of paternal allele identification in early pregnancy plasma samples.

Family	*CFTR* locus Mutation/Variant^A^	PGT	Week 5 gestation			Week 6 gestation			Week 7 gestation			Week 8 gestation			Final classification?^E^		APAiF^F^	NPTPpF^B^
			FL (%)	Results		FL (%)	Results		FL (%)	Results		FL (%)	Results					
				ASP-SEQ	TDS		ASP-SEQ	TDS		ASP-SEQ	TDS		ASP-SEQ	TDS	ASP-SEQ	TDS		
1	rs2237722	C/G	0.7	G	NR	2.3	G	NR	—	—	—	3.2	G	G	YES	YES	G	3
2	rs6466623^C^	T/C	—	—	—	—	—	—	—	—	—	0.3	NR	NR	NO	NO	T	1
3	rs2237726	C/T	0.8	NR	NR	1.2	NR	NR	—	—	—	—	—	—	NO	NO	C	2
4	E819X/WT (c.2455 G > T)	G/T	2	G	NR	2.5	G	NR	3	G	G	—	—	—	YES	YES	G (WT)	3
5	W1282X/WT (c.3846 G > A)	G/A	—	—	—	2.5	G	NR	4.1	G	NR	—	—	—	YES	NO	G (WT)	2
6	W1282X/WT (c.3846 G > A)	G/A	0.75	NR	G^D^	—	—	—	2.2	NR	NR	2.1	A	NR	YES	YES^D^	A (MUT)	3
7	delF508/WT (c.1521_1523delCTT)	CTT/-	0.75	NR	NR	—	—	—	—	—	—	—	—	—	NO	NO	CTT (WT)	1
8	rs2237726	C/T	—	—	—	0.4	NR	NR	0.5	T	T	0.9	NR	NR	YES	YES	T	3
9	rs17547485	T/G	—	—	—	—	—	—	—	—	—	1.1	T	NR	YES	NO	T	1
11	W1282X/WT (c.3846 G > A)	G/A	—	—	—	0.5	NR	NR	—	—	—	0.7	G	NR	YES	NO	G (WT)	2
13	rs34401510^C^	T/C	0.4	NR	NR	—	—	—	—	—	—	—	—	—	NO	NO	T	1

Abbreviations: WT, wild type; MUT, mutant; PGT, paternal genotype; APAiF, actual paternal allele in fetus; FL, fetal load calculated from sequencing data according to ref.^11^; TDS, targeted deep sequencing; NR, no result; NPTPpF, number of plasma time points per family.

^A^Variants are *CFTR* intragenic unless indicated otherwise. Nucleotide positions in the *CFTR* gene are according to GenBank NM_000492.3. Amino acid residues are according to NP_000483.3.

^B^Indicates number of plasma samples collected per pregnant couple.

^C^Variant is located within the *CFTR*-proximal *CTTNBP2* gene.

^D^Allelic classification for this sample, via TDS, was incorrect.

^E^Indicates whether classification of paternal allele was achieved, given the diagnostic method (ASP-SEQ or TDS) and NPTPpF.

^F^As determined by amniotic fluid testing of the fetus.

Overall, testing outcome for each couple in the study was heavily influenced by the number of plasma samples provided for evaluation. With ASP-SEQ, correct allelic inheritance was determined for 6 out of 7 couples who provided a minimum of 2 plasma samples for testing. For the seventh couple in this group (Family 3), allelic classification could not be determined, but importantly, there was no misdiagnosis (Table 1). On the other hand, in the one plasma sample group, a test result (albeit a correct amniotic fluid validated result) was obtained for just 1 out of 4 couples. Nonetheless, in this group too, there were no misdiagnoses (Table 1).

**Figure 3:**
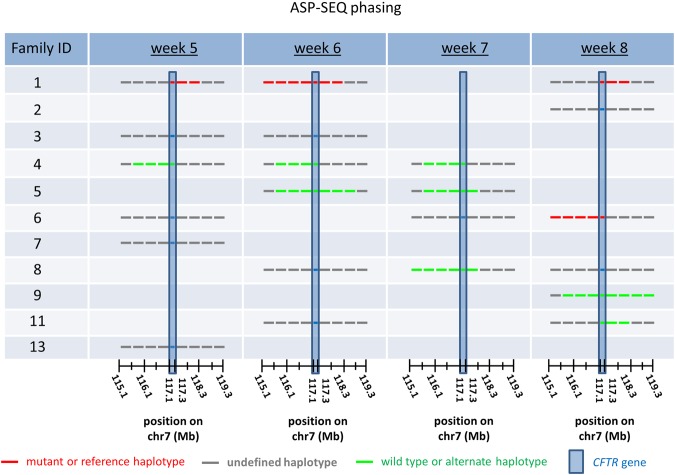
Paternal allele identification in early pregnancy plasma samples according to ASP-SEQ. Paternal haplotype block predictions in the *CFTR* gene-flanking region, according to ASP-SEQ (‘ASP-SEQ phasing’), are illustrated by Family ID and gestational age (in weeks) of the fetus at the time of plasma collection as in Fig. 2B. Families in which the father of the fetus was a *CFTR* mutation carrier received ‘mutant’ or ‘wild type’ assignments. Otherwise, *CFTR*-flanking haplotypes were assigned ‘reference’ designation when they matched that of an immediate family member used to establish paternal phase or ‘alternate’ designation when they did not. Other details regarding this series of experiments are summarized in Table 1. Note that in all samples, haplotype block predictions across the assayed genomic region were unambiguous with either defined mutant/wild type/reference/alternate designations or undefined designations in ASP-SEQ data. There were no conflicting ASP-SEQ haplotype assignments in each plasma sample-specific analysis.

Also of note is the fact that the fetal load in most plasma samples in the study was markedly low, with an average and median dosage of 1.5% and 1.0%, respectively. Nonetheless, despite the low overall fetal concentration per sample, 7 out of 11 couples obtained accurate (amniotic fluid validated) paternal allele classification in their respective fetuses by ASP-SEQ testing. Moreover, haplotype classification was remarkably clear and unambiguous using the ASP-SEQ method (see Fig. 3). Strikingly, ASP-SEQ also successfully diagnosed paternal alleles in the overall data from each early pregnancy time point (week 5; week 6; week 7; and week 8) suggesting that plasma from earlier weeks of gestation pose no greater challenge for ASP-SEQ than plasma from later weeks of gestation (Table 1).

**Figure 4:**
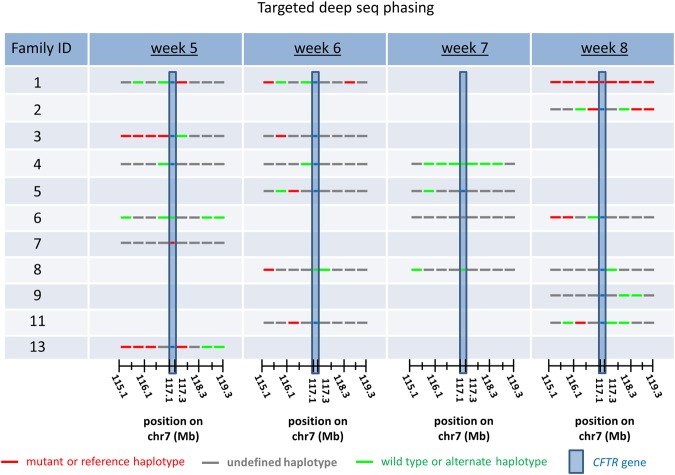
Paternal allele identification in early pregnancy plasma samples according to TDS. Paternal haplotype block predictions in the *CFTR* gene-flanking region, according to TDS (‘Targeted deep seq phasing’), are illustrated by Family ID and gestational age (in weeks) of the fetus at the time of plasma collection. Other details regarding this series of experiments are summarized in Table 1. Note that for most samples, haplotype block predictions across the assayed genomic region were largely inconsistent and ambiguous in TDS data.

Regarding TDS performance with the same early pregnancy cohort, the results were, as expected, far less accurate. TDS derived paternal allele test results for only 4 out of 7 couples in the two or more plasma sample group, one result of which was incorrect as determined by amniotic fluid testing (see Table 1, Family 6). In the one plasma sample group, TDS was unable to obtain a single result for any of the four couples (Table 1). These disappointing outcomes in the TDS data were not unexpected, as the 1.5% and 1.0% average and median fetal load dosages in the study were well below the accepted 4% fetal load threshold for standard NIPD sample processing. Accordingly, this crucial factor undoubtedly contributed to a majority of conflicting and confusing haplotype designations in the TDS data, in general (see Fig. 4). Moreover, there were 12 plasma samples from weeks 5 and 6 of pregnancy in the study and none of them provided a correct result after TDS assessment. The only successfully diagnosed paternal alleles, by means of TDS, were obtained from weeks 7 and 8 samples and, even at these time points only 3 out of 10 samples altogether were diagnosed (Table 1).

Therefore, in summary, these results strongly suggest that ASP-SEQ is the method of choice for very early pregnancy paternal allele detection in maternal blood despite markedly low fetal load dosage. Indeed, ASP-SEQ may be the only method capable of such ultra-sensitive allele detection with reproducible consistency.

## Discussion

We describe a novel, effective, and sensitive approach to very early pregnancy (week 5–8) NIPD of paternal alleles using *CFTR* analysis as a proof of principle. This approach may also be easily applied to fetal testing of any other disease-associated autosomal gene locus.

Specifically, the primary improvements set forward in this paper pertain to in-case test sensitivity. Therefore, we propose ASP-SEQ as a significant forward step in the development of early pregnancy diagnostic methods. Our new method is not more expensive and time-consuming than existing TDS and digital PCR NIPD methods, yet in terms of sensitivity and accuracy, it is far superior. Indeed, any method that affords couples the opportunity to assess their fetus’ genotype in very early pregnancy should be considered ground-breaking for the field of prenatal diagnostics because early diagnosis may afford previously unfeasible disease treatment/management options. Furthermore, the waiting period of expectant couples for results of the genetic status of their fetus is shortened and therefore overall stress of the prenatal testing process is decreased. Nonetheless, like most molecular tests, ASP-SEQ does not lack limitations. In *in vitro* testing, ASP-SEQ effectively isolated heavily diluted alleles down to 0.1% dosage (in family ‘A’) because the diluted alleles were controllably added to the specimen starting material. However, not all of the diluted paternal-specific SNP sites were detected by the test. Thus, direct ASP-SEQ of the mutation of interest could not be performed in lieu of the chosen haplotype-based method. Moreover, with actual plasma samples one cannot control whether any of the 0.1% dosed paternal allele informative SNP sites will actually enter the blood collection tube on the first blood draw. In fact, most of the couples in the study cohort who obtained a test result via ASP-SEQ had provided two or more blood draws. Theoretically, increasing the volume of blood collection at each blood draw (an option that was unavailable to the investigators in this study) may help to improve the ‘diagnosis rate’ (the percentage of plasma samples achieving diagnosis per blood draw) but proper vigilance in the clinical setting dictates that couples would be much better off if they provided at least 2 separate blood draws at 1 week intervals to ensure accurate and reportable test results. At the very least, blood drawing could be scheduled to coincide with early pregnancy follow up visits to the clinic with a β-HCG chemical pregnancy confirmation at week 5 and a clinical pregnancy confirmation by ultrasound during week 6. Also, to defray extra cost of dual sample processing per couple, sequencing libraries from both blood draws can be loaded onto the same high throughput sequencing run. Another limitation of ASP-SEQ is that it is not yet fit for maternal allele diagnosis during pregnancy. For this very important application (especially for autosomal recessive disorders such as CF), it will be crucial to identify highly definitive molecular biomarkers to distinguish maternal allele fetal DNA from maternal host DNA. There have been multiple discoveries in recent years regarding fetal biomarkers in maternal plasma including fetus-specific plasma DNA size^17–20^, methylation state^21–24^, and fragmentation pattern^19,25^; but these biomarkers are either too sparsely distributed along the human genome for ubiquitous mutation allele targeting or they are not quite conclusive enough for clinical utility in fetus-mother interrogation. In addition, one of the only reports to successfully describe early (week 5 gestation) pregnancy single gene testing^26^ has not yet been supported by other cases of early pregnancy diagnostic success. Therefore, more research is needed to improve fetal biomarker discovery going forward. In the meantime, the current ASP-SEQ method may readily be applied to any paternal dominant disorder; or to any autosomal recessive disorder as an ‘exclusion test’ to determine whether a father had transmitted his wild type allele to a fetus. For the latter application subsequent invasive/noninvasive prenatal tests to ascertain the mother’s allelic status in the fetus might be avoided^27^.

Thus, it is not in the distant future when NIPD will become available for very early pregnancy assessment of monogenic diseases. The appropriate tools and ultra-sensitive methods are already in place to ensure that pregnant couples never run out of options at each and every stage and milestone of pregnancy.

## Methods

### Sample collection and DNA extraction

Couples undergoing preimplantation genetic diagnosis (PGD) at the Shaare Zedek Medical Center (SZMC) PGD or Assaf Harofeh IVF clinics were recruited into the study. Pregnant study participants and their partners provided at least one or more peripheral blood samples between weeks 5 and 8 of gestation. For pregnant female indices, plasma was separated from peripheral blood by centrifugation at 1,900 × *g* for 10 minutes at 4 °C. The plasma supernatant was then recentrifuged at 16,000 × *g* for 10 minutes at 4 °C and 3 ml of the resulting supernatant was used for cell-free DNA extraction with the QIAamp Circulating Nucleic Acid kit (QIAGEN) according to the manufacturer’s protocol. The maternal plasma DNA extracts were then pre-amplified, in duplicate, with the NEBNext® Ultra™ II DNA Library Prep kit (New England Biolabs) ahead of downstream processing. The inclusion criteria for the investigation were as follows: singleton clinical pregnancy had to be confirmed by ultrasound during week 6 gestation; the couple’s first degree family member genomic DNA samples needed to be available for parental haplotype phasing purposes; a DNA sample from CVS or amniotic fluid testing from a later stage of pregnancy had to be provided during the course of the study for test validation purposes. Couples who did not meet all of the study’s inclusion criteria were excluded from the investigation and not analyzed by any genetic testing methods. Generally, there was preference to recruit PGD pregnant couples into the study who were carriers of *CFTR* mutations. However, in the PGD clinic, most couples refrain from performing follow up invasive prenatal testing to confirm the genetic status of their fetus due to high PGD accuracy rates and fear of miscarriage of a “very precious” pregnancy. Hence, CF-mutation carriage was not required from study participants who each signed informed consent to allow their plasma samples to be analyzed for non-pathogenic *CFTR*-proximal single nucleotide polymorphisms (SNPs). For the non CF couples, all underwent screening for at least 14 common mutations in the *CFTR* gene prior to the study.

### Next generation sequencing (NGS) of CFTR-flanking single nucleotide polymorphisms (SNPs)

Custom targeted deep sequencing (TDS) and ASP-SEQ panels were designed to sequence and genotype 1,700 *CFTR*-flanking SNPs (with minor allerle frequency>25%). However, only ASP-SEQ panels were designed to sequence SNP targets in an allele-specific manner. TDS panels sequenced SNP targets without any allele-specificity. Accordingly, the TDS panel was applied to genotype all samples (genomic and plasma DNA) in the study while the ASP-SEQ panel was applied to plasma and genomic maternal DNA samples only. For both TDS and ASP-SEQ panels, indexed next generation sequencing libraries were prepared and normalized according to the manufacturer’s protocol (Illumina) followed by 2 × 150 pair-end sequencing on a MiSeq or NextSeq. 500 instrument (Illumina) to a mean depth of 1000x for genomic and plasma DNA samples. After sequencing runs, the data were aligned to target sequences on the human reference genome (hg19) and genotyping data was extracted from each alignment and annotated using GATK software^28^. These profiles were then combined into single family-specific.csv files so as to facilitate familial and fetal linkage analysis (see below). As a rule, paternal haplotypes were constructed with SNPs for which the father of the fetus was heterozygous and at least one of his/her first degree relatives was homozygous.

### Standard haplotype construction and identification of fetal paternal alleles in maternal plasma DNA using TDS

For each genomic DNA sample in the study (whether from the pregnant couple or their first degree family members), heterozygous genotype calls from TDS were trio-phased to obtain paternal allele-specific haplotypes. TDS was then used to identify paternal mutations, variants, or *CFTR*-flanking alleles in all plasma samples as previously described^11^.

### Identification of fetal paternal alleles in maternal plasma DNA using ASP-SEQ

ASP-SEQ was used to identify paternal mutations, variants, or *CFTR*-flanking alleles in all plasma samples by comparing ASP-SEQ results of maternal genomic DNA with its corresponding plasma DNA ASP-SEQ libraries. For every maternal genomic and accompanying plasma DNA sample with standard deep sequencing genotype information, two different targeted ASP-SEQ libraries were prepared. ASP-Seq_A_ libraries amplified only reference SNP alleles (“A”) but not non-reference alleles (“B”). Conversely, ASP-Seq_B_ libraries amplified only non-reference SNP alleles (“B”) but not reference alleles (“A”). After high throughput sequencing of each ASP-SEQ library, successfully amplified regions were mapped to the human genome and utilized to detect fetal DNA that did not exist in maternal only genomic DNA. Thus, for every fetal haplotype informative SNP locus, ASP-SEQ determined whether a “child-specific” allele was transmitted to the fetus or not. In parallel, TDS was performed on paternal genomic DNA and paternal family member genomic DNA to determine if the “child-specific” alleles existed also in a particular paternal haplotype. Plasma DNA samples were sequenced in duplicate at high depth (>1,000x mean coverage) and only paternal haplotype informative SNPs (father heterozygote and mother homozygote) were analyzed. Paternal haplotype informative SNPs feature a unique nucleotide in the fetus’ father that is not present in the maternal genotype. All other parental SNP combinations were not utilized for ASP-SEQ-based paternal allele derivation. Paternal haplotype informative SNPs were assessed from a minimum read depth of 100x whereupon only allele-specific amplification of the paternal “unique allele” in the plasma ASP-SEQ libraries that did not appear in maternal genomic DNA ASP-SEQ library controls were incorporated into the fetal haplotype. This filter was applied so as to reduce genotyping errors emerging from either sequencing error and/or off-target sequence contamination.

Ultimately, fetal diagnosis was achieved after comparing the paternal cell-free fetal DNA (cffDNA) haplotypes with family-based trio phase haplotypes as relevant. Altogether, the entire noninvasive NGS-based prenatal test, from blood sample processing to fetal diagnosis, was completed in 5 work days. In addition, all diagnoses were confirmed by prenatal amniotic fluid genetic testing.

### Ethics approval, consent to participate, and method accordance

Ethical approval for the study, including usage of materials from human subjects, was obtained from the local SZMC institutional review board (SZMC-134/12) and written informed consent was obtained from all study participants. The methods described herein were carried out in accordance with relevant guidelines and regulations.

## Data Availability

The datasets used and/or analysed during the current study are available from the corresponding author on reasonable request.
